# 3*H*-1,2-Dithiole-3-thione Suppresses LPS-induced Proinflammatory Responses in Macrophages: Potential Involvement of Antioxidant Induction, NF-κB, and Nrf2

**DOI:** 10.1007/s11010-021-04331-x

**Published:** 2022-02-18

**Authors:** Hong Zhu, An Bui, Arben Santo, Y. Robert Li

**Affiliations:** 1 Campbell University Jerry M. Wallace School of Osteopathic Medicine, Buies Creek, NC 27506, USA; 2 Edward Via College of Osteopathic Medicine, Virginia Tech Corporate Research Center, Blacksburg, VA 24060, USA

**Keywords:** 3*H*-1,2-Dithiole-3-thione, RAW 264.7 cells, NF-κB, Nrf2, IL-1β, TNF-α

## Abstract

Previously, we reported that 3*H*-1,2-dithiole-3-thione (D3T), an Nrf2 activator, acted as a potential chemoprotectant against lipopolysaccharide (LPS)-induced mortality in mice. In view of the critical involvement of macrophages in the pathogenesis of LPS-induced endotoxemia, in the present study, we investigated the protective effects of D3T on LPS-induced proinflammatory responses in cultured murine RAW 264.7 macrophage cell line and primary peritoneal macrophages and the potential involvement of antioxidant induction, NF-κB, and Nrf2. We showed that treatment with D3T resulted in increased levels of a series of antioxidants in RAW 264.7 cells in a concentration-dependent manner. These included the reduced form of glutathione (GSH), glutathione peroxidase, glutathione reductase, glutathione *S*-transferase, and NADPH:quinone oxidoreductase 1. Catalase was also potently induced by D3T which, however, did not show a concentration dependency. Concurrent with the ability to induce the above cellular antioxidants, D3T pretreatment of RAW 264.7 cells also led to a concentration-dependent suppression of LPS-induced interleukin-1beta (IL-1β) production and nitric oxide release. LPS-stimulated tumor necrosis factor-alpha (TNF-α) production was also suppressed by D3T, but to a much lesser extent. Using NF-κB reporter gene-expressing RAW 264.7 cells, we further showed that D3T pretreatment also suppressed LPS-induced NF-κB activation. To investigate the potential involvement of Nrf2, a chief regulator of cellular antioxidant genes, we used peritoneal macrophages isolated from Nrf2^+/+^ and Nrf2^−/−^ mice. Our results showed that D3T pretreatment suppressed LPS-induced proinflammatory responses in Nrf2^+/+^ macrophages, and this inhibitory effect of D3T was completely lost in Nrf2^−/−^ macrophages. Collectively, the results of the present study demonstrated that D3T acted as a potent suppressor of LPS-induced proinflammatory responses in macrophages. Antioxidant induction, NF-κB suppression, and Nrf2 activation appeared to contribute to the anti-proinflammatory activity of D3T in macrophages.

## 1. Introduction

Endotoxemia and sepsis remain a major cause of death worldwide, accounting for nearly 20% of all global deaths [1]. Although cytokine storm and dysregulated inflammation have long been recognized as a major pathophysiological mechanism of sepsis, effective therapies for sepsis remain lacking [2–4]. Hence, identification of critical molecular and cellular targets of sepsis and development of mechanistically based modalities for sepsis intervention are of paramount clinical significance. In this context, recent studies have demonstrated a critical role for oxidative stress, NF-κB, and Nrf2 signaling in the pathophysiology of endotoxemia and sepsis in animal models [5, 6].

Dithiolethiones are five-membered cyclic sulfur-containing compounds, some of which are constituents of cruciferous vegetables [7]. We and others have found that 3*H*-1,2-dithiole-3-thione (D3T) is the most potent member of dithiolethiones for the induction of endogenous antioxidative/anti-inflammatory and phase 2 enzymes in various types of tissues and cells, including hepatic tissue [7, 8], myocardium [9], bone marrow stromal cells [10], and macrophages [11], among others. As oxidative and inflammatory stress are intimately involved in the pathophysiology of various diseases, upregulation of these cellular defenses by D3T has been shown to protect against such disease processes as chemical carcinogenesis [12], neurodegeneration [13], and cardiovascular injury [14] in experimental models. Notably, we and others have also previously demonstrated that D3T-mediated upregulation of antioxidative/anti-inflammatory defenses occurs via activation of Nrf2, the chief regulator of cytoprotective genes [8, 14, 15]. Indeed, D3T is among the most potent activators of Nrf2 signaling and the activation of Nrf2 is indispensable for D3T-mediated cytoprotection against oxidative and electrophilic injury [10, 15, 16]. Recently, our work further suggested that D3T also protected against lipopolysaccharide (LPS)-induced mortality in mice in an Nrf2-dependent manner [17]. D3T, like other Nrf2 activators, may thus be developed as a potential chemoprotectant for sepsis intervention. To this end, further elucidation of the molecular processes underlying D3T-mediated anti-proinflammatory responses in cells is warranted. As macrophages are crucial inflammatory cells involved in cytokine storm and inflammatory syndrome [18], in the present study, we used murine macrophage line RAW 264.7 cells and primary peritoneal macrophages isolated from Nrf2^+/+^ and Nrf2^−/−^ mice as in vitro models to investigate the protective effects of D3T on LPS-induced proinflammatory responses and the potential involvement of antioxidant induction, NF-κB, and Nrf2 in D3T-mediated anti-proinflammation.

## 2. Materials and Methods

### 2.1. Materials

D3T with a purity of 99.8% was generously provided by Dr. Mary Tanga at SRI International (Menlo Park, CA) and Dr. Linda Brady at National Institute of Mental Health (Bethesda, MD). RPMI-1640 medium, penicillin, streptomycin, fetal bovine serum (FBS), and Dulbecco’s phosphate buffered saline (PBS) were from Thermo Fisher Scientific (Waltham, MA). All other chemicals and agents were from Sigma-Aldrich (St. Louis, MO). Cell culture flasks and other plastic wares were purchased from Thermo Fisher Scientific (Waltham, MA).

### 2.2. Cell cultures

Regular RAW 264.7 cells were obtained from ATCC (Manassas, VA) and NF-κB reporter gene-expressing RAW 264.7 cells were purchased from Genlantis (San Diego, CA). Primary peritoneal macrophages were isolated from inhouse-bred male Nrf2^+/+^ and Nrf2^−/−^ C57BL/6 mice as described previously following the procedures approved by Institutional Animal Care and Use Committee [11]. All cells were cultured in RPMI-1640 medium supplemented with 10% FBS, 100 units/ml of penicillin, and 100 μg/ml of streptomycin at 37°C in a humidified atmosphere of 5% CO_2_. For measuring D3T-mediated induction of cellular antioxidants, the cells cultured on tissue culture flasks were incubated with D3T (0, 25, 50, and 100 μM) at 37°C for 24 h. To determine the effects of D3T on LPS-induced cytokine and nitric oxide release, cells were pretreated with D3T for 24 hours and then exposed to LPS (10 ng/ml) for another 24 h followed by the measurement of the endpoints released into culture media. On the other hand, activation of NF-κB was measured after a 6-h exposure to LPS. Cellular protein content was quantified with Bio-Rad protein assay dye (Hercules, CA) using bovine serum albumin as the standard.

### 2.3. Measurement of cellular antioxidants

Preparation of cell extracts and measurement of cellular levels/activities of reduced glutathione (GSH), glutathione peroxidase (GPx), glutathione reductase (GR), glutathione *S*-transferase (GST), catalase, and NADPH:quinone oxidoreductase 1 (NQO1) were performed according to the published procedures described by us before [10, 11, 19].

### 2.4. Measurement of IL-1β and TNF-α

The levels of IL-1β and TNF-α released into the culture media were measured by using corresponding mouse Quantikine ELISA kits from R&D Systems (Minneapolis, MN) according to the manufacturer’s instruction. A concurrently run standard curve was used for quantification.

### 2.5. Measurement of nitric oxide

The production of nitric oxide was determined by measuring the total amount of nitrate/nitrite released into the culture media by using a commercial kit from R&D Systems (Minneapolis, MN) according to the manufacture’s instruction. A concurrently run standard curve was used for quantification.

### 2.6. Determination of NF-κB activation

The cells expressing the NF-kB-luciferase report gene were lysed, and the luciferase activity was measured using a Berthold LB9505 multichannel luminometer (Wildbad, Germany). The intensity of the luminescence is proportional to the degree of NF-kB activation.

### 2.7. Statistical analysis

All data are expressed as means ± standard deviation (SD) from at least three separate experiments unless otherwise indicated. Differences between mean values of multiple groups were analyzed by one-way analysis of variance followed by Student-Newman-Keuls test. Differences between two groups were analyzed by Student’s t test. Statistical significance was considered at p < 0.05.

## 3. Results and Discussion

### 3.1. D3T-mediated induction of antioxidants in RAW 264.7 cells

**Fig 1.**
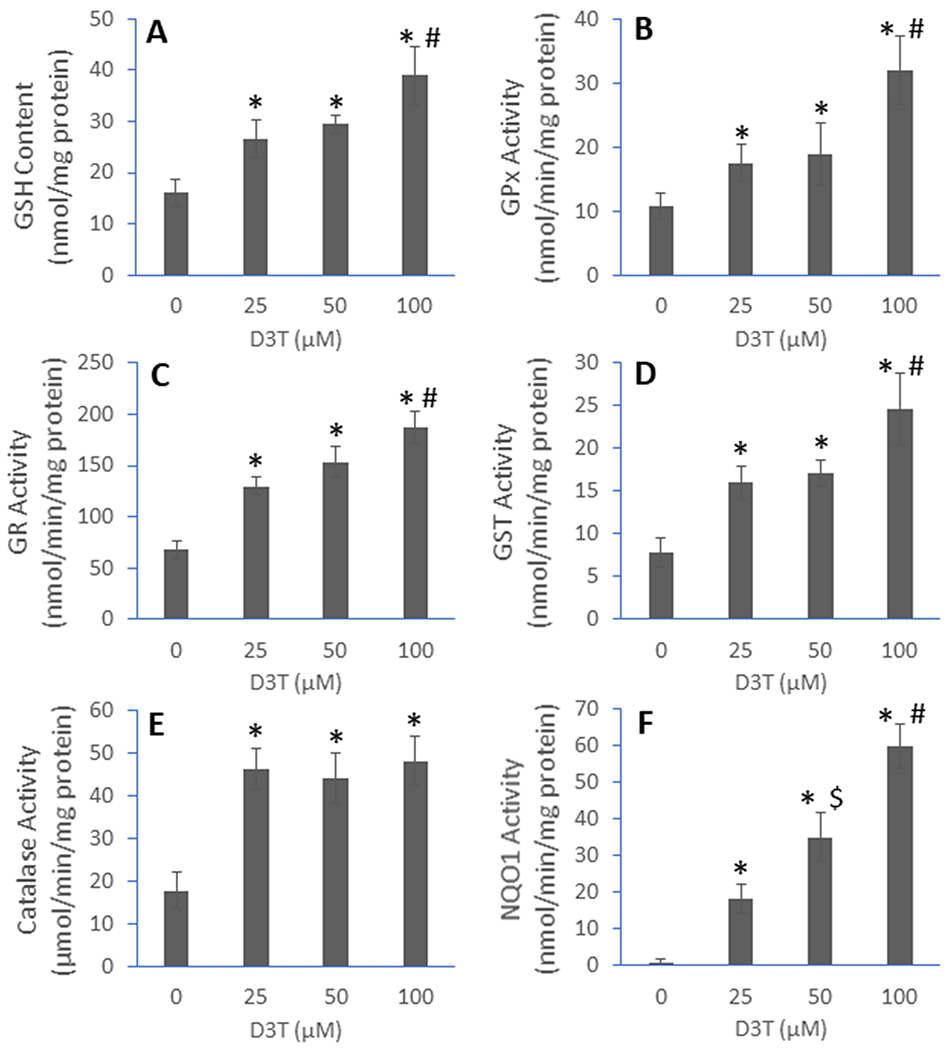
D3T-mediated induction of antioxidants in RAW 264.7 cells. The cells were incubated with the indicated concentrations of D3T (0, 25, 50, and 100 μM) for 24 h followed by measurement of the levels or activities of various cellular antioxidants, including GSH (A), GPx (B), GR (C), GST (D), catalase (E), and NQO1 (F). Data represent mean ± SD (n = 4-8). *, p < 0.05 versus 0 μM D3T; #, p < 0.05 versus 25 and 50 μM D3T; $, p < 0.05 versus 25 μM D3T.

We previously showed that D3T induced a host of cellular antioxidant defenses in primary peritoneal macrophages isolated from mice [11]. For the present study, we first determined if D3T also induced antioxidants in RAW 264.7 cells, a mouse macrophage cell line. As shown in Figure 1, incubation of RAW 264.7 cells with D3T for 24 h resulted in significant induction in cellular GSH, GPx, GR, GST, catalase, and NQO1. The induction of the above antioxidants, except for catalase, showed a D3T-concentration dependency. It remains unclear why catalase induction was independent of D3T concentrations used (25-100 μM). It is possible that 25 μM D3T was enough to cause the maximal induction of catalase under the present experimental conditions.

GSH, GPx, GR, and GST are members of the glutathione system—a major cellular antioxidant defense system involved in the detoxification of various reactive oxygen and nitrogen species (ROS/RNS) [20]. On the other hand, catalase is an enzyme selective for decomposing hydrogen peroxide, whereas NQO1 acts as both a quinone-detoxifying enzyme and a superoxide scavenger [21]. As ROS/RNS are critical players in dysregulated inflammatory responses [22], induction of the above cellular antioxidants would render macrophages more resistant to oxidative stress and dampen macrophage’s proinflammatory responses. Thus, this antioxidant induction experiment would set a stage for the subsequent investigation of the effects of D3T on LPS-induced proinflammatory responses.

### 3.2. D3T-mediated suppression of LPS-induced proinflammatory responses in RAW 264.7 cells

**Fig 2.**
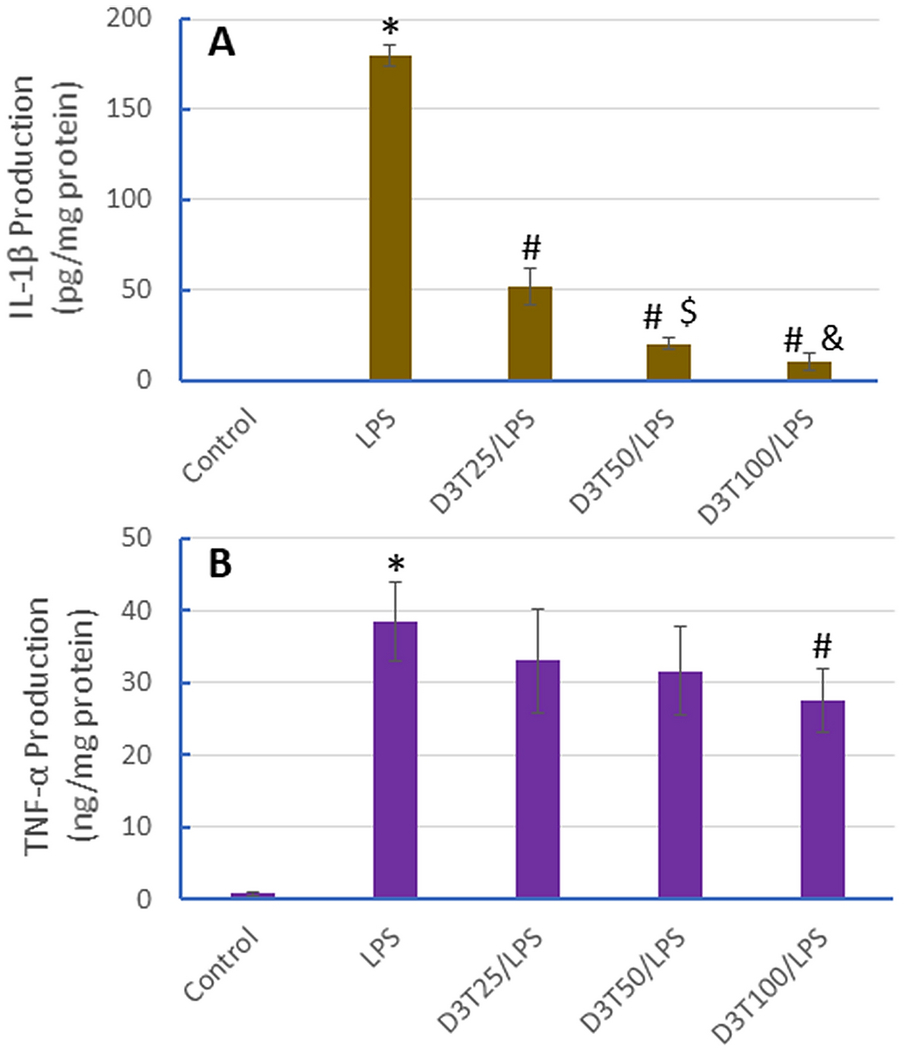
D3T-mediated suppression of LPS-induced proinflammatory cytokine release in RAW 264.7 cells. The cells were pretreated with the indicated concentrations of D3T (0, 25, 50, and 100 μM) for 24 h and then exposed to LPS (10 ng/ml) for another 24 h, followed by measurement of IL-1β (A) and TNF-α (B) release into the culture media. Data represent mean ± SD (n = 4-6). *, p < 0.05 versus Control (0 μM D3T); #, p < 0.05 versus LPS only; $, p < 0.05 versus D3T 25 μM/LPS; &, p < 0.05 versus D3T 50 μM/LPS.

Release of proinflammatory cytokines by macrophages has been shown to play a critical role in many inflammatory disorders, especially the acute phase of sepsis [21, 23]. We next determined if D3T pretreatment could suppress LPS-induced production of IL-1β and TNF-α, two major proinflammatory cytokines, in macrophages. As shown in Figure 2, under the basal unstimulated condition, RAW 264.7 cells released minimal amounts of IL-1β and TNF-α; however, exposure of the cells to LPS led to dramatically increased release of both IL-1β and TNF-α into the culture media. Notably, pretreatment of the cells with D3T resulted in a marked concentration-dependent attenuation of LPS-induced IL-1β release. A 71%, 89%, and 94% reduction in LPS-induced IL-1β release was seen with 25, 50, and 100 μM D3T pretreatment, respectively (Figure 2A). In contrast, the effect of D3T pretreatment on LPS-induced TNF-α release was much less remarkable; only the highest concentration of D3T caused a 28% reduction (Figure 2B). This differential effect on IL-1β versus TNF-α suggests that different signaling pathways may be involved in the LPS-induced release of these two proinflammatory cytokines. Indeed, a previous study showed that blocking mitochondrial ROS production blunted LPS-induced IL-1β expression without affecting LPS-induced TNF-α expression [18].

**Fig 3.**
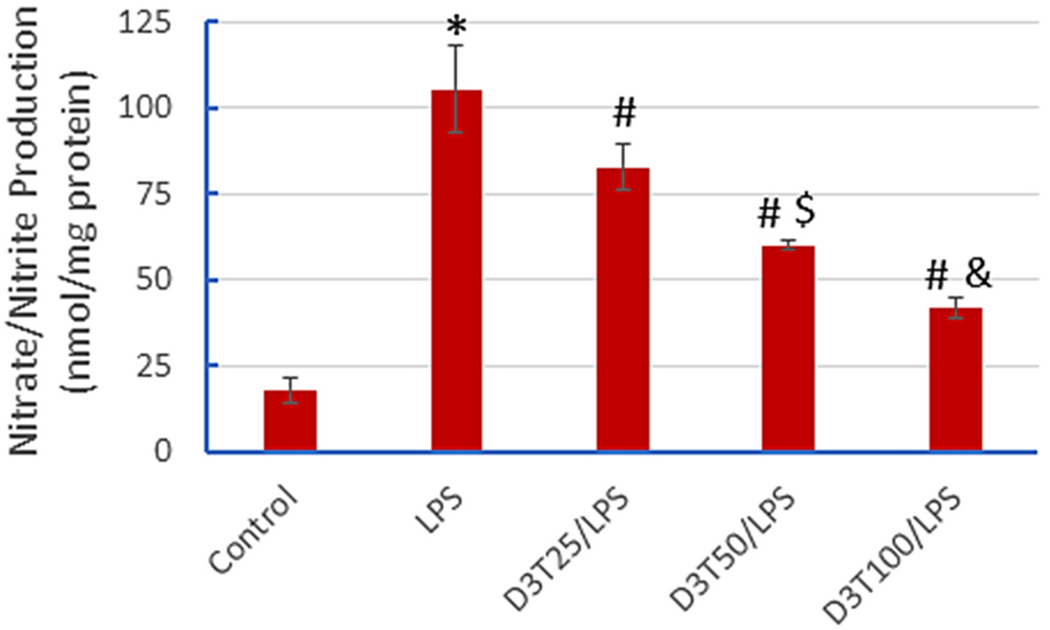
D3T-mediated suppression of LPS-induced nitric oxide release in RAW 264.7 cells. The cells were pretreated with the indicated concentrations of D3T (0, 25, 50, and 100 μM) for 24 h and then exposed to LPS (10 ng/ml) for another 24 h, followed by measurement of nitric oxide (in the form of nitrate/nitrite) release into the culture media. Data represent mean ± SD (n = 3). *, p < 0.05 versus Control (0 μM D3T); #, p < 0.05 versus LPS only; $, p < 0.05 versus D3T 25 μM/LPS; &, p < 0.05 versus D3T 50 μM/LPS.

To investigate the effect of D3T pretreatment on ROS/RNS production, we measured the total nitric oxide production by RAW 264.7 cells. In line with the effects on the proinflammatory cytokines, LPS exposure dramatically increased the production of total nitric oxide (as assessed by measuring nitrate/nitrite formation) in the cells. The LPS-induced RNS production was significantly blunted by D3T pretreatment in a concentration-dependent manner (Figure 3). It remains unclear how D3T pretreatment reduced LPS-induced nitric oxide production. It is well established that LPS activates inducible nitric oxide synthase (iNOS), leading to increased production of nitric oxide in macrophages [24, 25]. The suppression by D3T on LPS-induced nitric oxide production in RAW 264.7 cells might result from two potential mechanisms: (i) scavenging nitric oxide by the D3T-induced cellular antioxidants (e.g., GSH) and (ii) suppression of LPS-induced NF-κB activation (see Section 3.3). In this context, LPS-induced iNOS expression occurs via an NF-κB-dependent mechanism [26].

### 3.3. D3T-mediated suppression of LPS-induced NF-κB activation in RAW 264.7 cells

**Fig 4.**
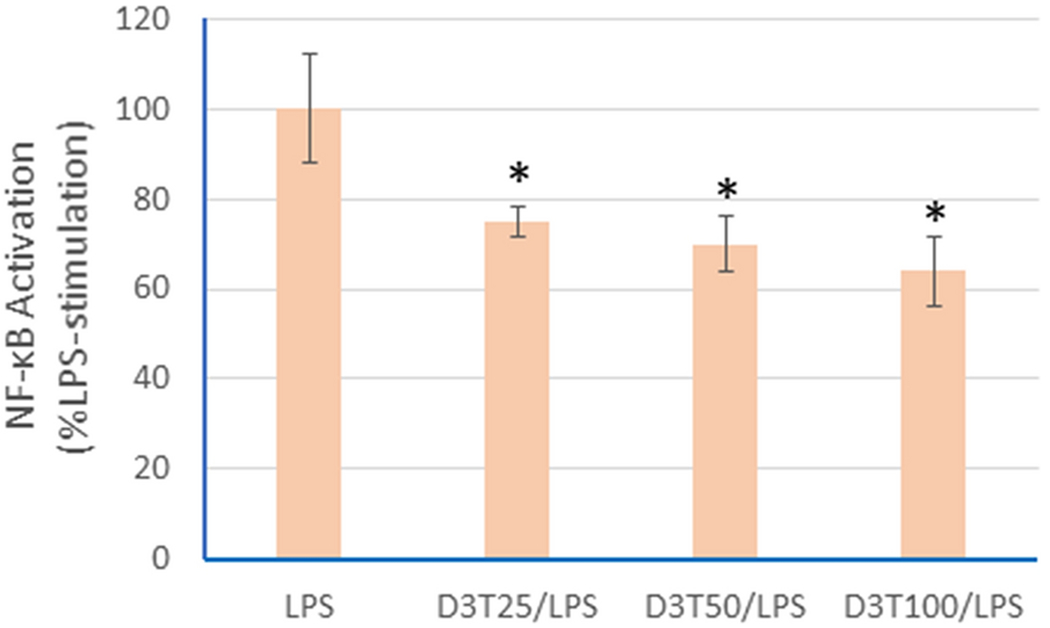
D3T-mediated suppression of LPS-induced NF-κB activation in RAW 264.7 cells. The cells were pretreated with the indicated concentrations of D3T (0, 25, 50, and 100 μM) for 24 h and then exposed to LPS (10 ng/ml) for another 6 h, followed by detection of NF-κB activation. Data represent mean ± SD (n = 4). *, p < 0.05 versus LPS only.

Since NF-κB is a chief regulator of the gene expression of proinflammatory cytokines (including IL-1β and TNF-α) as well as iNOS [27], we determined if D3T pretreatment could also suppress LPS-induced NF-κB activation. As shown in Figure 4, the LPS-stimulated NF-κB activation was significantly suppressed by D3T pretreatment. However, the degree of NF-κB suppression by D3T was not in line with the extent of D3T-mediated IL-1β suppression (Figure 2A). In this regard, the suppression of LPS-induced NF-κB activation by D3T did not show a concentration dependency and only a 25-35% inhibition was seen with D3T (25-100 μM) pretreatment (Figure 4). On the other hand, LPS-induced IL-1β production was suppressed by 71-94% following D3T pretreatment. This discrepancy suggests that inhibition of NF-κB signaling is only one of the mechanisms of D3T-mediated blockage of LPS-induced IL-1β production in macrophages.

### 3.4. D3T-mediated suppression of LPS-induced proinflammatory responses in Nrf2^+/+^ and Nrf2^−/−^ peritoneal macrophages

**Fig 5.**
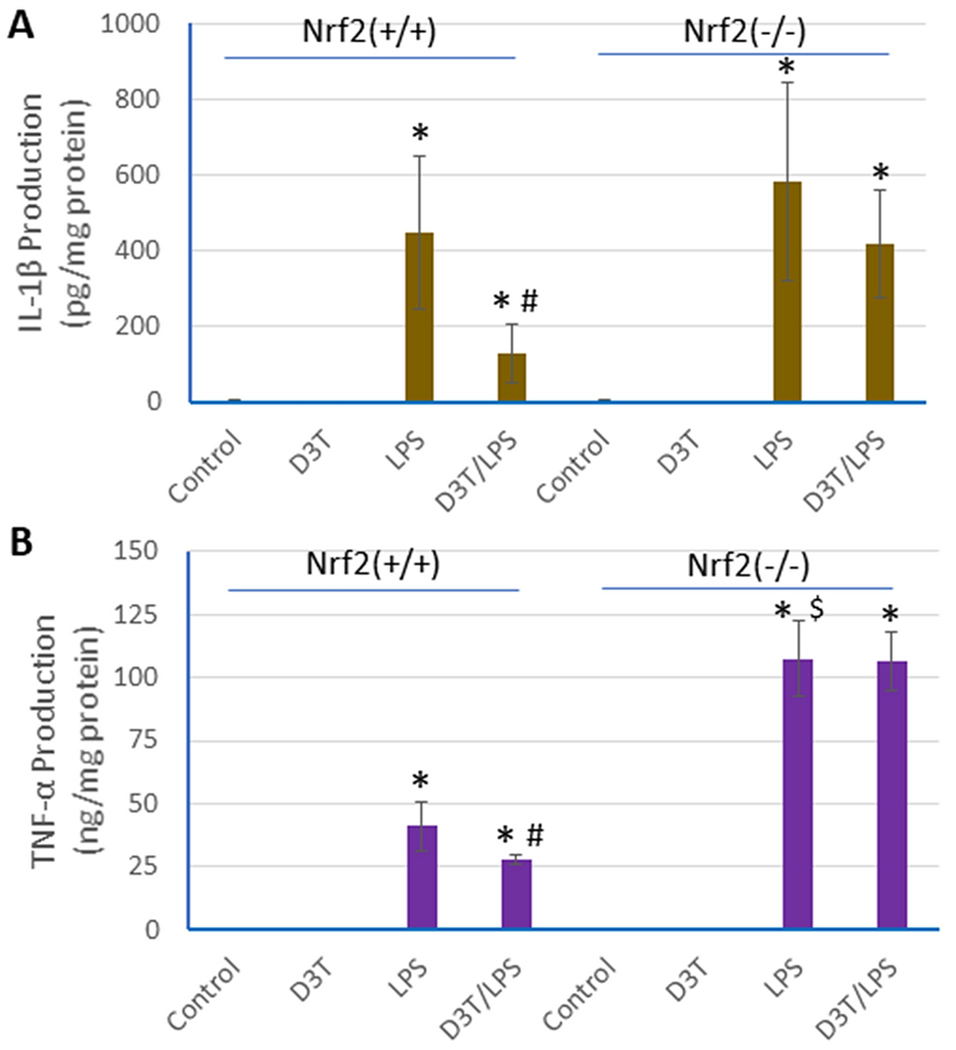
D3T-mediated suppression of LPS-induced proinflammatory responses in Nrf2^+/+^ and Nrf2^−/−^ peritoneal macrophages. Peritoneal macrophages of Nrf2^+/+^ and Nrf2^−/−^ genotypes were pretreated with 0 (control) and 100 μM D3T for 24 h and then exposed to LPS (10 ng/ml) for another 24 h, followed by measurement of IL-1β and TNF-α release into the culture media. Data represent mean ± SD (n = 3). *, p < 0.05 versus respective Control; #, p < 0.05 versus LPS only; $, p < 0.05 versus LPS only in Nrf2^+/+^ cells.

We previously showed that D3T-mediated induction of antioxidants in macrophages was dependent on Nrf2 status [11] and D3T is a potent activator of Nrf2 as well as an inducer of Nrf2 expression [17]. Accordingly, we determined if Nrf2 status could also affect the ability of D3T to suppress LPS-induced proinflammatory cytokine release. To this end, we used peritoneal macrophages isolated from Nrf2-null (Nrf2^−/−^) mice and the wild-type (Nrf2^+/+^) mice. As shown in Figure 5, the suppressing effect of D3T pretreatment on LPS-induced IL-1β and TNF-α release was completely lost in the Nrf2^−/−^ macrophages, suggesting that Nrf2 signaling is indispensable for the anti-proinflammatory activity of D3T in macrophages. Notably, the absence of Nrf2 resulted in a drastic augmentation of the LPS-induced release of TNF-α (Figure 5B); there was also an increasing trend in LPS-induced IL-1β release, but it did not reach statistical significance due to large sample variations (Figure 5A). Nevertheless, this finding suggests that the inducible express of proinflammatory cytokines, especially TNF-α, could readily become out of control in the absence of functional Nrf2 signaling, causing overt inflammatory stress. Indeed, targeted disruption of Nrf2 gene has been shown to dramatically sensitize animals to inflammatory tissue injury in an experimental sepsis animal model [17, 28].

**Fig 6.**
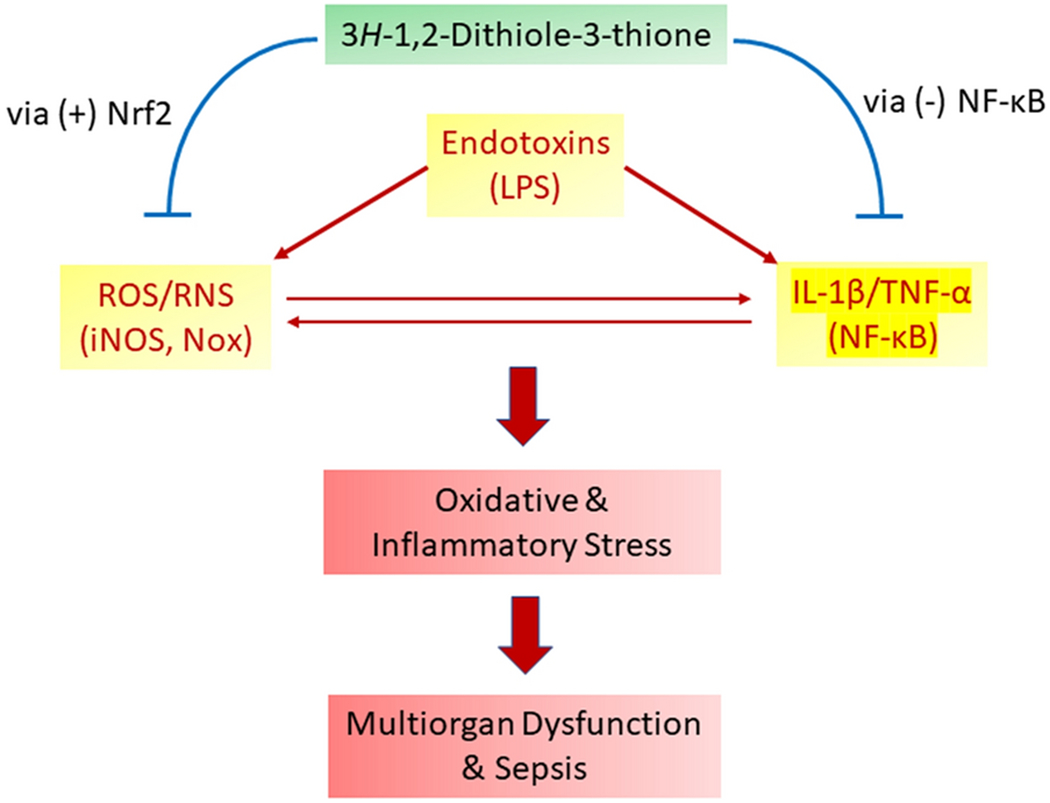
Schematic illustration of the potential involvement of Nrf2 activation (antioxidant induction) and NF-κB inhibition in D3T-mediated suppression of oxidative and inflammatory stress underlying endotoxemia and sepsis. Nox denotes NADPH oxidase, a well-established ROS-producing enzyme stimulated by LPS.

In conclusion, the results of the present study demonstrated that D3T—a cruciferous dithiolethione-related compound, is effective in attenuating LPS-induced proinflammatory stress in macrophages, and Nrf2-dependent antioxidant induction as well as suppression of NF-κB activation may collectively contribute to the ability of D3T to protect against experimental sepsis observed previously in mice [17] (Figure 6). It should be noted, however, that the present study did not focus on delineating the detailed signaling pathways on which D3T may act to suppress LPS-induced proinflammatory responses. In fact, the exact signaling pathways involved in LPS-induced cytokine (e.g., IL-1β) expression remain to be defined. In this context, recent studies by others showed that LPS-induced IL-1β production was dependent, at least partly, on mitochondrial ROS production [18, 29]. Hence, the ability of D3T to increase intracellular and intramitochondrial antioxidants [30] may account, at least partly, for the attenuation of LPS-induced IL-1β production.

It should also be noted that D3T may exert its effects independent of Nrf2 activation. In this regard, D3T has been shown to react with sulfhydryl groups [31] and may thus directly modify cellular proteins including those involved in LPS receptor signaling. Nevertheless, the dramatic abolishment of D3T-mediated protective effects on LPS-induced proinflammatory responses in Nrf2-null macrophages suggests that Nrf2 activation is a major underlying mechanism.

A final notion about the findings of the present study is that we previously demonstrated that D3T induces a battery of antioxidant and phase 2 enzymes in macrophages via upregulating specific isozyme gene expression as evidenced by increased mRNA and protein levels of the specific isozymes [11]. As such, in the present study, we did not determine the mRNA or protein expression of the antioxidant isoenzymes. Moreover, the exact contribution of the individual Nrf2-regulated antioxidant enzymes to D3T-mediated suppression of LPS-induced proinflammatory responses in macrophages remains to be determined. In this context, an early study suggested that selective overexpression of NQO1 in human monocytes attenuated LPS-induced TNF and IL-1β expression [32]. As efficient detoxification of ROS/RNS requires the complementary actions of various cellular antioxidant enzymes, we propose that the coordinated upregulation of diverse cellular antioxidants by D3T via an Nrf2-dependent mechanism may represent a much more effective strategy than overexpression of a single antioxidant enzyme in protecting against oxidative and inflammatory stress underlying endotoxemia and sepsis (Figure 6).

## Data Availability

The datasets generated during and/or analyzed during the current study are available from the corresponding author on reasonable request.
